# Advancing Allogeneic NK Cell Immunotherapy through Microfluidic Gene Delivery

**DOI:** 10.1002/advs.202412544

**Published:** 2025-03-07

**Authors:** Hyelee Kim, Mujin Lee, Bohwa Han, Jinho Kim, Duck Cho, Junsang Doh, Aram J. Chung

**Affiliations:** ^1^ Department of Bioengineering Korea University Seoul 02841 Republic of Korea; ^2^ Interdisciplinary Program in Precision Public Health (PPH) Korea University Seoul 02841 Republic of Korea; ^3^ Department of Materials Science and Engineering Seoul National University Seoul 08826 Republic of Korea; ^4^ Department of Health Sciences and Technology SAIHST Sungkyunkwan University Seoul 06355 Republic of Korea; ^5^ Department of Laboratory Medicine and Genetics Samsung Medical Center Sungkyunkwan University School of Medicine Seoul 03063 Republic of Korea; ^6^ School of Biomedical Engineering Korea University Seoul 02841 Republic of Korea; ^7^ MxT Biotech Seoul 04785 Republic of Korea

**Keywords:** allogeneic cell therapy, CAR‐NK, CRISPR, gene delivery, microfluidics, NK cell‐based immunotherapy

## Abstract

Chimeric antigen receptor (CAR)‐T cell therapy has revolutionized cancer treatment, yet challenges such as manufacturing complexity, high costs, and safety concerns have spurred the development of alternatives like CAR‐natural killer (NK) cell immunotherapies. CAR‐NK cell therapies provide innate cytotoxicity with antigen‐independent targeting, reducing safety risks while improving therapeutic efficacy. However, efficient genomic engineering and large‐scale production of allogeneic NK cells remain significant obstacles. To address these challenges, a novel microfluidic gene delivery platform is developed, the Y‐hydroporator, designed for allogeneic NK cell immunotherapy. This platform features a Y‐shaped microchannel where NK cells experience rapid hydrodynamic stretching near the stagnation point, creating transient membrane discontinuities that facilitate the uptake of exogenous cargo. The Y‐hydroporator achieves high delivery and transfection efficiency, processing ≈2 × 10^6^ cells min^−1^ while maintaining long‐term cell viability (>89%) and functionality. Using this platform, human primary CAR‐NK cells and NKG2A‐knockout NK cells are successfully generated by delivering anti‐CD19 CAR mRNA and CRISPR/Cas9 ribonucleoproteins, respectively. These engineered NK cells demonstrated enhanced cytotoxicity, underscoring the potential of the Y‐hydroporator as a transformative tool for advancing allogeneic NK cell‐based immunotherapies.

## Graphical abstract

The Y‐Hydroporator is a microfluidic gene delivery platform designed for allogeneic NK cell‐based immunotherapies. It achieves highly efficient gene delivery while preserving high cell viability and functionality. By producing engineered NK cells with enhanced cytotoxicity, the platform addresses critical limitations of autologous cell therapy, providing a robust and scalable tool for advancing next‐generation cancer immunotherapies.

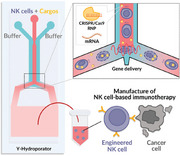

## 1 Introduction

Chimeric antigen receptor (CAR)‐T cell therapies^[^ ^1^ ^]^ have transformed cancer treatment by demonstrating substantial efficacy against hematological malignancies, including lymphoblastic leukemia,^[^ ^2^ ^]^ B‐cell lymphoma,^[^ ^3^ ^]^ and multiple myeloma.^[^ ^4^ ^]^ CARs are synthetic receptors engineered to enable T cells to recognize and eliminate cancer cells expressing specific antigens, such as CD19.^[^ ^5^ ^]^ Unlike natural T cell receptors, CARs interact with target antigens independently of the major histocompatibility complex (MHC) class I, resulting in robust T cell activation and potent antitumor responses.^[^ ^6^ ^]^ The impact of CAR‐T cell therapy is underscored by the U.S. Food and Drug Administration (FDA) approval of six CAR‐T cell products^[^ ^7^ ^]^ and numerous ongoing clinical trials worldwide.^[^ ^8^ ^]^

Despite its promise, CAR‐T cell therapy faces significant limitations, primarily due to the complex engineering required to modify patient T cells. The manufacturing process begins with isolating T cells from peripheral blood mononuclear cells (PBMCs) via leukapheresis, followed by viral transduction to introduce the CAR‐encoding gene. This process is time‐consuming, costly,^[^ ^9^ ^]^ and prone to a manufacturing failure rate of ≈10% due to conditions such as lymphopenia and T‐cell exhaustion.^[^ ^10^ ^]^

To address these challenges, allogeneic CAR‐T cells derived from healthy donor T cells have emerged as a viable alternative.^[^ ^11^ ^]^ These cells are typically engineered by knocking out αβ T cell receptors, which mediate antigen specificity, using genomic editing techniques.^[^ ^12^ ^]^ However, infusion of allogeneic CAR‐T cells can lead to complications such as graft‐versus‐host disease and cytokine release syndrome, both of which can be life‐threatening.^[^ ^11^ ^]^

A promising alternative to CAR‐T cell therapy involves the use of natural killer (NK) cells, which are not antigen‐specific.^[^ ^13^ ^]^ NK cells naturally target cancerous and infected cells through a balance of activating and inhibitory receptors.^[^ ^14^ ^]^ By combining tumor antigen‐specific CARs with healthy NK cells, the therapeutic efficacy can be enhanced, leveraging both innate and adaptive immune responses. This “off‐the‐shelf” allogeneic NK cell therapy addresses the limitations of autologous CAR‐T therapies and has shown comparable efficacy in treating CD19‐positive lymphoid tumors.^[^ ^15^ ^]^

The manufacturing process for CAR‐NK cells, similar to CAR‐T cells, begins with isolating CD56‐positive NK cells from PBMCs.^[^ ^9^ ^]^ The subsequent critical step is introducing the CAR‐encoding gene into activated primary NK cells, typically via viral transduction or electroporation. Viral transduction effectively achieves CAR expression on the cell surface but is limited by high costs, lengthy production times,^[^ ^16^ ^]^ and safety concerns related to oncogenic and mutagenic risks.^[^ ^17^ ^]^ Additionally, in NK cells, viral transduction can trigger antiviral responses, leading to apoptosis.^[^ ^18^ ^]^ Electroporation, an alternative gene delivery method, uses electrical energy to transiently disrupt the cellular membrane for exogenous gene internalization. While it is not restricted by cell or cargo type and avoids issues related to innate immunity,^[^ ^16b^ ^]^ the high electrical energy involved often causes irreversible genomic damage^[^ ^19^ ^]^ and cytotoxicity.^[^ ^20^ ^]^

In response to these drawbacks, various gene delivery approaches for CAR‐NK cell manufacturing are under active investigation.^[^ ^21^ ^]^ Among these, microfluidic techniques have demonstrated significant potential in addressing the associated challenges.^[^ ^16b^ ^]^ Microfluidic systems offer precise spatiotemporal control over fluid flow and cells, miniaturization, reduced sample consumption, and scalability, making them particularly appealing for the intracellular delivery of genetic material into cells.^[^ ^22^ ^]^ One common microfluidic approach involves passing cells through narrow constrictions in microchannels to temporarily permeabilize the cell membranes for biomolecule internalization. However, this constriction‐based mechanoporation technique is hindered by issues such as channel clogging and low transfection efficiency for plasmid DNA.^[^ ^22f,g^ ^]^ Other platforms that rely on microscale fluidic phenomena, such as vortex shedding,^[^ ^22h^ ^]^ have also been proposed, but they exhibit relatively low delivery and transfection efficiency.

To overcome these limitations, we developed a novel microfluidic gene delivery platform tailored for NK cell‐based immunotherapy. The platform, termed the Y‐hydroporator, is specifically designed to process human primary NK cells. By leveraging the elongation of NK cells during flow to facilitate the internalization of exogenous cargo, this system achieves high delivery/transfection efficiency while maintaining long‐term cell viability and functionality. This makes it a robust tool for the production of CAR‐NK cells. The Y‐hydroporator's capacity to enable allogeneic NK cell‐based immunotherapy offers a promising solution to the current limitations of CAR‐T cell therapies.

## 2 Results and Discussion

### 2.1 Platform Design, Working Principle, and Validation

Cell mechanoporation involves the disruption of cell membranes to facilitate the intracellular delivery of external cargo.^[^ ^16b^ ^]^ Recently, we introduced a microfluidics‐based delivery platform leveraging fluid‐cell interactions to internalize external macromolecules into cells.^[^ ^20a^ ^]^ While the platform demonstrated effective delivery of various nanomaterials, such as quantum dots, nanospheres, mRNA, and plasmid DNA, its performance was inadequate for NK cell‐based immunotherapy and genome editing applications. We hypothesized that enhancing fluid‐cell interactions by modifying the channel architecture and delivery scheme (described below) would improve its effectiveness for NK cell engineering.

**Figure 1:**
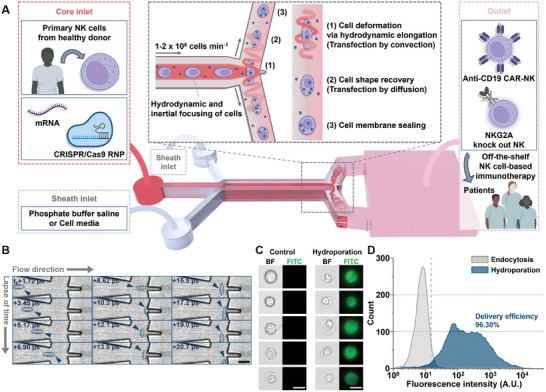
Device design and working principle. A) Schematic representation of the manufacturing process for NK cell‐based immunotherapies using the Y‐junction hydroporator. B) High‐speed microscopy images showing the hydrodynamic elongation of NK cells. C) Microscopy images illustrating bright‐field (BF) and FITC fluorescence for 2000 kDa FITC‐dextran internalization via endocytosis (Control) and hydroporation. D) Fluorescence intensity histograms displaying the delivery of 2000 kDa FITC‐dextran into NK cells (*N*_cell_ = 5000 cells per sample). All scale bars: 10 µm.

To address this, we designed a Y‐junction hydroporator, as illustrated in **Figure** **1**A. Although similar in appearance to our previous platform, the new design incorporates critical modifications to enhance NK cell transfection efficiency without requiring additional cargo while supporting long‐term operation. The Y‐hydroporator includes three inlets: one core inlet and two sheath inlets. Cells mixed with cargo (e.g., mRNA or CRISPR/Cas9 systems) were injected through the core inlet, while regular cell media is introduced through the sheath inlets. Upon injection, cells were inertially positioned at the channel center with controlled spacing,^[^ ^23^ ^]^ ensuring uniform mechanoporation downstream. At the Y‐junction stagnation point, cells underwent instantaneous elongation (≈20 µs) due to helical recirculating flows within the microchannel (see Figure 1A(1),B; Movie S1, Supporting Information). Upon migrating downstream, the cells restored their original spherical shape. This rapid deformation and recovery induced convective internalization of exogenous macromolecules through nanopores formed in the membrane. These openings resealed within 1–2 min,^[^ ^21^ ^]^ further allowing small cargo to diffuse into cells before closure (Figure 1A(2),(3)).

To validate the Y‐hydroporator's effectiveness in delivering cargo to primary NK cells, we tested the internalization of fluorescein isothiocyanate (FITC)‐conjugated dextran (2000 kDa) at 0.3 mg mL^−1^. FITC‐dextran was selected due to its minimal cell surface binding, attributed to its negative charge. After 18 h of hydroporation, fluorescence microscopy confirmed strong fluorescence exclusively in NK cells processed with the Y‐hydroporator (Figure 1C). FITC‐dextran was detected in both the cytosol and nucleus, confirming successful and uniform nuclear delivery. Quantitative delivery assessment via flow cytometry (see Experimental Section for details) revealed that NK cells processed with the Y‐hydroporator achieved a substantially higher delivery efficiency of 96.3% compared with the positive control group, which accounted for endocytosis and surface interactions (Figure 1D). These results validate the platform's potential for advanced applications in primary NK cell engineering.

### 2.2 Optimization of Y‐Hydroporator

**Figure 2:**
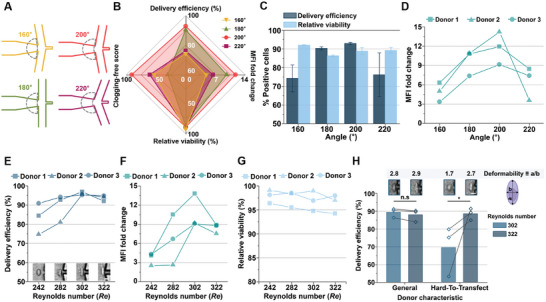
Optimization of microfluidic intracellular delivery. A) Schematic of junction angles ranging from 160° to 220°. B) Delivery efficiency, MFI fold change, relative viability, and clogging‐free score for 2000 kDa FITC‐dextran delivery into NK cells at different junction angles. C) Delivery efficiency and relative viability of 2000 kDa FITC‐dextran delivery into NK cells across various junction angles (*N* = three independent donors). D) MFI fold change for 2000 kDa FITC‐dextran delivery into NK cells with different junction angles. E) Delivery efficiency of 2000 kDa FITC‐dextran into NK cells at varying Reynolds numbers (*Re*). Subsets: Representative high‐speed images of cell deformation at different *Re* values. F) MFI fold change for 2000 kDa FITC‐dextran delivery into NK cells across various *Re* values. G) Relative viability of NK cells following delivery of 2000 kDa FITC‐dextran at varying *Re* values. H) Delivery efficiency of 2000 kDa FITC‐dextran into NK cells and cell deformability based on donor characteristics and *Re* values (*N* = three independent donors). All bars represent mean ± standard deviation (SD). *P < 0.05 indicates a significant difference, while n.s denotes no significant difference. Student's t‐test was used to compare two experimental groups. All scale bars: 10 µm.

An ideal gene delivery platform for cell‐based therapy must process large numbers of cells while delivering functional macromolecules efficiently and cost‐effectively to meet clinical demands. Although increasing cell concentration can improve scalability, we observed that high cell density in a T‐junction channel often reduced delivery efficiency due to channel clogging.^[^ ^20^, ^22^ ^]^ To address this, we hypothesized that a Y‐shaped channel design could reduce clogging by facilitating smoother flow toward the outlet while also enhancing cell deformation to improve delivery efficiency. To test this hypothesis, we evaluated microchannels with junction angles of 180° and 200°. The 180° channel design (**Figure** **2**A) builds on our previous work,^[^ ^22a^ ^]^ while the 200° design incorporates modifications described in detail below. Using high‐speed microscopy, we assessed cell deformability as an indicator of intracellular delivery potential.^[^ ^22b^ ^]^ As shown in Figures 1B and S1 (Supporting Information), the 200° junction angle increased cell deformability compared to the 180° design, suggesting an improved potential for delivery.

Before assessing precise delivery efficiency and the impact of clogging at different junction angles, we examined the cargo injection scheme for the cell‐cargo mixture. The Y‐hydroporator's three‐inlet layout allowed testing of three distinct cargo injection setups: cargo introduced through the core inlet only (Figure S2A(1), Supporting Information), through the sheath inlets only (Figure S2A(2), Supporting Information), or through both the core and sheath inlets simultaneously (Figure S2A(3), Supporting Information). We observed significantly higher delivery efficiency and increased mean fluorescence intensity (MFI) fold change when the cargo was introduced through the core inlet alone (Figure S2B, Supporting Information). This outcome was attributed to the cargo introduced into the sheath fluids being carried toward the outlet rather than trapped in vortices.^[^ ^22a^ ^]^ Based on these findings, we adopted the core stream injection strategy for subsequent experiments. To further reduce operational costs, we replaced the sheath fluid with a cost‐effective buffer, such as phosphate‐buffered saline (PBS), without compromising delivery efficiency (data not shown).

Additionally, we decreased the core media flow rate to minimize cargo usage. While this reduced the cell processing rate, we compensated by increasing cell concentration to meet throughput requirements and adjusted the sheath flow rate to maintain consistent flow‐cell interactions, ensuring a constant Reynolds number (*Re*). This approach was validated by testing two cases, both of which maintained equivalent delivery efficiency while reducing cargo usage and achieving comparable cell processing rates (Figure S2C,D, Supporting Information).

With the implementation of these new cargo injection and flow rate adjustment schemes, we evaluated delivery efficiency, cell viability, MFI fold change, and clogging scores (see Experimental Section) for junction angles ranging from 160° to 220° (Figure 2A). Using 2000 kDa FITC‐dextran for delivery into NK cells from three independent donors (Figure 2B–D), we found that channels with 180° and 200° angles achieved high delivery efficiency (≈90%). The 200° junction angle demonstrated the highest MFI fold change, consistent with our findings on cell deformability (Figure S1, Supporting Information). This enhanced deformation likely arises from the larger momentum transfer of fluid particles along the x‐axis in the 200° design, compared to the sharp 90° turn required in the 180° channel. Notably, the 220° junction angle, which was expected to enhance delivery due to the larger angle, resulted in cells exiting the outlet instead of being trapped in vortices for delivery (see Movie S2, Supporting Information).

Next, we investigated clogging as a function of the junction angle. The 160° junction angle exhibited the least cell clogging overall; however, small debris, such as polydimethylsiloxane (PDMS) fragments and dust, became lodged in the cavity, causing significant clogging. Conversely, the 220° junction angle design led to elongated cells stacking over the cavity, resulting in channel blockage. The 180° junction angle exhibited a combination of clogging behaviors similar to those observed at 160° and 220°. In contrast, the 200° junction angle achieved a nearly clogging‐free score (≈100), making it the optimal design for minimizing clogging.

We also evaluated flow rates to optimize delivery performance. Higher flow rates improved delivery efficiency and MFI fold change but reduced cell viability. To identify ideal operational conditions, we measured MFI fold change, delivery efficiency, and cell viability at different flow rates (i.e., *Re*). With the core flow rate fixed at 100 µL min⁻¹ to minimize cargo consumption (as shown in Figure S2C,D, Supporting Information), the sheath flow rate was systematically adjusted. When delivering 2000 kDa FITC‐dextran into NK cells from three independent donors, increased sheath flow rates enhanced delivery efficiency (Figure 2E) and MFI fold change (Figure 2F), corresponding to greater hydrodynamic elongation of NK cells (Figure 2E subsets). However, excessive elongation at higher *Re* values reduced relative viability (Figure 2G), thereby lowering overall delivery efficiency. The optimal *Re* for intracellular delivery into NK cells was determined to be 302, achieving over 90% delivery efficiency and an MFI fold change of approximately nine while maintaining high cell viability and preventing clogging.

All results in this study were based on tests using human primary NK cells, with data collected from at least three independent donors. Interestingly, we observed a variability in delivery efficiency among NK cells, with some subsets exhibiting notably lower delivery efficiency under the same conditions. This variability was attributed to donor‐specific heterogeneity among NK cells. To investigate further, we analyzed the deformability of low‐efficiency NK cells. As shown in Figure 2H, these subsets displayed reduced deformability (≈1.7) compared to the general donor population (≈2.7). Increasing the sheath flow rate to *Re* = 322 improved the deformability of these less‐deformable NK cells (≈90%), matching the levels observed in the general population. Based on these findings, we established two tailored flow conditions for hydroporation of primary human NK cells, depending on their deformability characteristics.

The optimal experimental conditions were identified as a *Re* of 302 (or 322 for less‐deformable subtypes), a fixed core flow rate of 100 µL min⁻¹, a channel junction angle of 200°, and exclusive addition of all cargo to the core stream. These conditions were applied in all subsequent delivery experiments, as described in the following sections.

### 2.3 Intracellular Delivery Characterization of Various Primary Immune Cells

**Figure 3:**
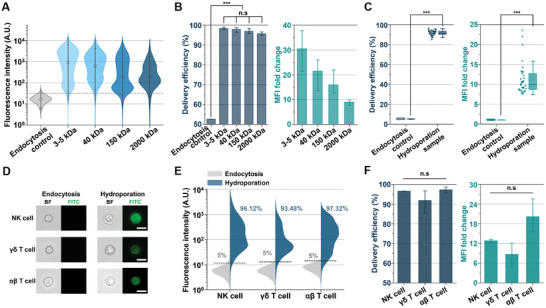
Intracellular delivery characterization of human primary NK, γδ T, and αβ T cells. A) Fluorescence intensity histograms showing the delivery of FITC‐dextran of varying sizes into NK cells (*N*_cell_ = 5000 per sample). B) Delivery efficiency and MFI fold change for FITC‐dextran of various sizes delivered into NK cells (*N* = three independent donors). C) Delivery efficiency and MFI fold change for 2000 kDa FITC‐dextran delivery into NK cells (*N* = 35 independent donors). D) Microscopy images showing bright‐field and FITC fluorescence for 2000 kDa FITC‐dextran transfer via endocytosis (Control) or hydroporation into NK, γδ T, and αβ T cells. Scale bars: 10 µm. E) Fluorescence intensity histograms illustrating the delivery of 2000 kDa FITC‐dextran into NK, γδ T, and αβ T cells (*N*_cell_ = 5000 per sample). F) Delivery efficiency and MFI fold change for 2000 kDa FITC‐dextran delivery into NK, γδ T, and αβ T cells (*N* = three independent donors). All bars represent mean ± SD. ***P < 0.001 indicates a highly significant difference, while n.s denotes no significant difference. Multiple comparisons were conducted using one‐way ANOVA. Student's t‐test was used to compare two experimental groups.

To characterize the efficiency of our microfluidic intracellular delivery platform in delivering various nanomaterials into NK cells under the identified operational conditions, we internalized differently sized FITC‐dextran molecules (3–5, 40, 150, and 2000 kDa) into NK cells. These experiments used a final FITC‐dextran concentration of 0.3 mg mL⁻¹ and a cell density of 1 × 10⁷ cells mL⁻¹. As shown in **Figure** **3**A, the Y‐hydroporator consistently achieved delivery efficiencies greater than 90% across different donor samples, significantly outperforming the endocytosis control group. Although the MFI fold change decreased with increasing FITC‐dextran size, hydroporated NK cells exhibited stronger fluorescence intensity compared to the control group (Figure 3B). We processed NK cells from 35 independent donors and consistently achieved a delivery efficiency of ≈90% (Figure 3C).

To evaluate the broader applicability of our platform, we tested γδ T cells, which are promising candidates for off‐the‐shelf allogeneic cell therapies due to their MHC class I‐independent cytotoxicity against cancer or infected cells.^[^ ^24^ ^]^ Additionally, we assessed αβ T cells, which are widely used in allogeneic CAR‐T cell therapies.^[^ ^11^ ^]^ For these experiments, we delivered 2000 kDa FITC‐dextran into activated and expanded γδ and αβ T cells at a cell density of 1 × 10⁷ cells mL⁻¹. Fluorescence microscopy images taken 18 h post‐delivery revealed distinct fluorescent signals in the hydroporated immune cells compared to the control group (Figure 3D). Flow cytometry analysis confirmed that all three immune cell types achieved delivery efficiencies exceeding 90% (Figure 3E). This high delivery efficiency was consistently observed across immune cells from three independent donors (Figure 3F). Notably, the MFI fold change for αβ T cells was significantly higher than that achieved in our previous study using a T‐junction hydroporator.^[^ ^20a^ ^]^ This improvement was attributed to the optimized channel design and the updated cargo injection configuration.

These results demonstrate the versatility of the Y‐hydroporator in effectively processing diverse cargo sizes and immune cell types, highlighting its potential for applications in allogeneic cell therapies.

### 2.4 Engineering NK Cells without Affecting Viability and Phenotype

**Figure 4:**
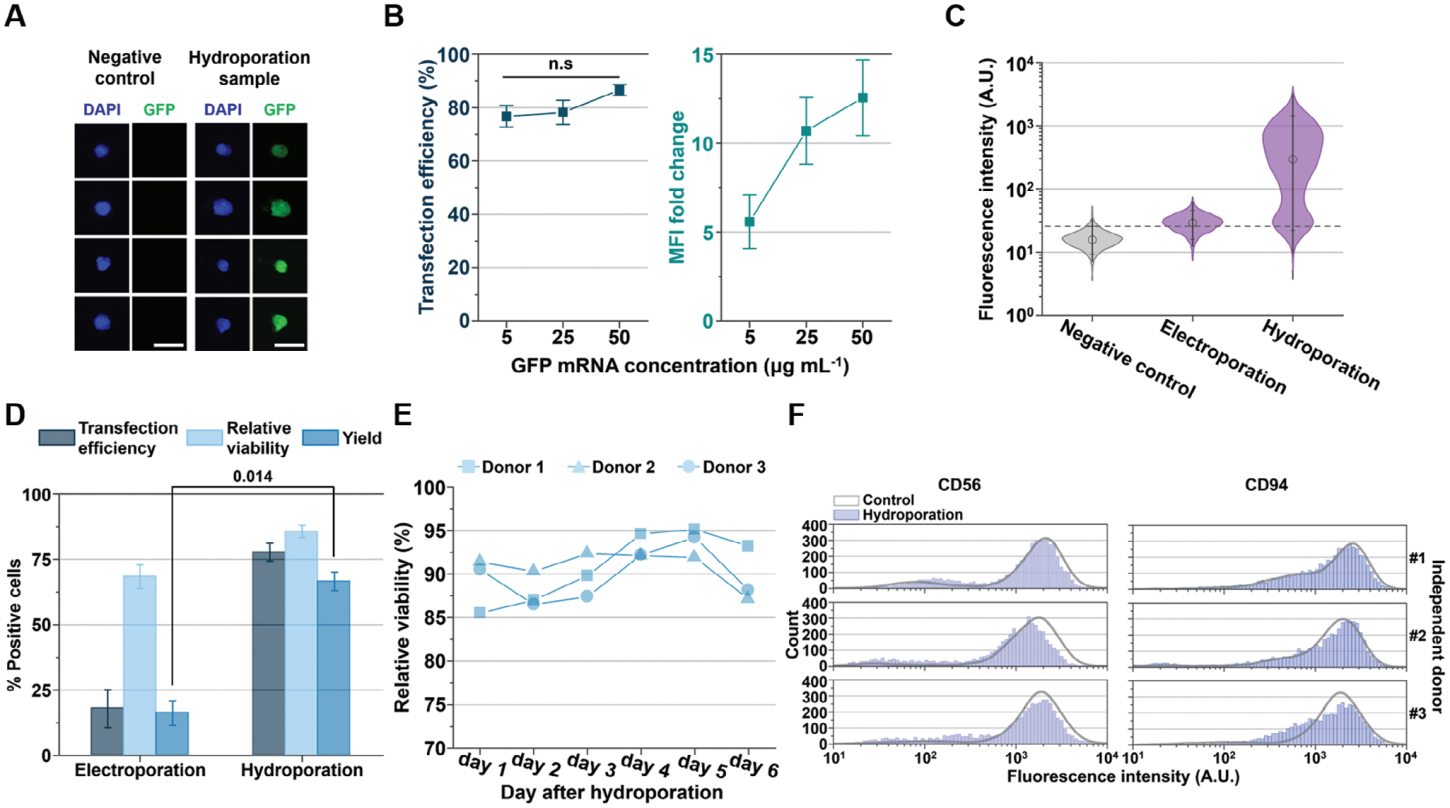
mRNA NK cell engineering and validation of cell viability and phenotype preservation. A) Confocal microscopy images of NK cells transfected with EGFP mRNA (25 µg mL^−1^), stained with 4’,6‐diamidino‐2‐phenylindole (DAPI) for nuclear visualization (scale bars: 10 µm). B) Transfection efficiency and MFI fold change for EGFP mRNA transfection in NK cells with varying mRNA concentrations (*N* = three independent donors). C) Fluorescence intensity histograms of EGFP mRNA transfection in NK cells at a concentration of 25 µg mL^−1^ via electroporation or hydroporation (*N*_cell_ = 5000 per sample). D) Transfection efficiency, MFI fold change, and NK cell yield for EGFP mRNA transfection using electroporation or hydroporation (*N* = three independent donors). E) Relative viability of hydroporated NK cells assessed with 7‐AAD staining over 6 days. F) Fluorescence intensity histograms of CD56 and CD94 immunostaining with phycoerythrin (PE)‐conjugated antibodies for the negative control and hydroporated NK cells after 24 h (*N*_cell_ = 5000 per sample). All bars represent mean ± standard deviation (SD). Student's *t*‐test was used to compare the two experimental groups. n.s denotes no significant difference. Multiple comparisons were conducted using one‐way ANOVA. Student's t‐test was used to compare two experimental groups.

Achieving effective transfection with functional nanomaterials encoding specific genes is essential for advancing NK cell‐based immunotherapy. To validate mRNA transfection using the Y‐hydroporator, we employed EGFP mRNA at concentrations of 5 to 50 µg mL⁻¹, based on previous studies.^[^ ^25^ ^]^ The mRNA was mixed with NK cells (1 × 10⁷ cells mL⁻¹) and injected through the core inlet. Following hydroporation, the mRNA‐transfected NK cells were incubated for 24 h to allow EGFP translation. NK cells treated with the Y‐hydroporator exhibited stronger fluorescence than those in the control group (**Figure** **4**A). Quantitative analysis of transfection efficiency and MFI fold changes showed that hydroporated NK cells achieved ≈80% transfection efficiency at a concentration of 5 µg mL⁻¹ (Figure 4B), surpassing previously reported values.^[^ ^20^, ^22^ ^]^ Increasing the mRNA concentration to 50 µg mL⁻¹ resulted in transfection efficiencies of ≈90% and a 12‐fold increase in MFI, indicating successful mRNA transfection.

Electroporation is currently one of the leading non‐viral transfection methods for NK cells, offering operational simplicity and relatively high editing efficiency.^[^ ^26^ ^]^ To compare the performance of the Y‐hydroporator with electroporation, we processed NK cells using 25 µg mL⁻¹ of EGFP mRNA. NK cells treated with the Y‐hydroporator exhibited significantly higher fluorescence than those treated with electroporation, indicating more effective mRNA transfection (Figure 4C). To assess net transfection effectiveness, we introduced a new metric, yield, defined as the product of delivery efficiency and cell viability. As shown in Figure 4D, the Y‐hydroporator achieved a significantly higher yield (≈60%) compared to electroporation, underscoring its potential as a viable alternative for NK cell‐based immunotherapies.

In addition to achieving high transfection yield, maintaining the viability and functionality of engineered NK cells is critical for effective cell‐based therapies.^[^ ^20a^ ^]^ We evaluated the relative viability of NK cells from three donors after hydroporation. Using 7‐aminoactinomycin D (7‐AAD) staining at 24‐hour intervals, we confirmed that NK cell viability remained consistently high (>85%) and was not adversely affected by hydroporation (Figure 4E). However, viability declined by day six, likely due to the natural lifespan of NK cells following expansion.

Next, we assessed the functionality of hydroporated NK cells by immunostaining for biomarkers such as CD56 and CD94, 24 h post‐treatment.^[^ ^20^, ^22^ ^]^ CD56 serves as a crucial biomarker for distinguishing NK cell subsets based on their immune response,^[^ ^27^ ^]^ while CD94 plays a central role in NK cell activation and inhibition, functioning as a core component of receptors such as NKG2A, NKG2C, and NKG2E.^[^ ^28^ ^]^ Flow cytometry histograms showed no significant differences in biomarker expression between non‐processed and hydroporated cells (Figure 4F), indicating that the phenotype and functionality of NK cells were well preserved. Recent RNA sequencing and cytokine release assays of human primary T cells processed via mechanoporation revealed no substantial alterations,^[^ ^20a^ ^]^ suggesting that NK cells also maintain genetic stability following hydroporation.

### 2.5 Production of CAR‐NK Cells using the Y‐Hydroporator

**Figure 5:**
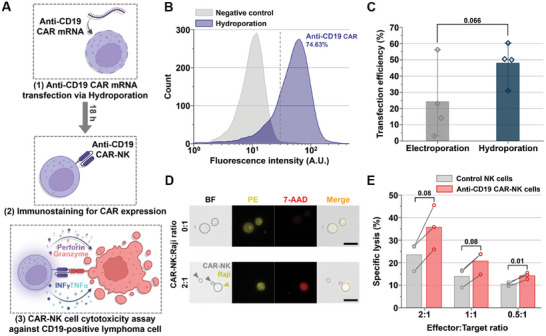
Production of anti‐CD19 CAR‐NK cells using the Y‐hydroporator. A) Schematic of CAR‐NK cell production. B) Fluorescence intensity histograms showing PE‐conjugated CD19 antigen immunostaining for the negative control and hydroporated NK cells after 18 h (*N*_cell_ = 5000 per sample). C) Transfection efficiency of anti‐CD19 CAR mRNA into NK cells via hydroporation and electroporation (*N* = four independent donors). D) Microscopy images of bright‐field and fluorescence showing PE and 7‐AAD staining, indicating Raji cells and cell death, respectively (scale bars: 25 µm). E) Specific lysis of CD19‐positive Raji cells co‐incubated with control NK cells or anti‐CD19 CAR‐NK cells (*N* = three independent donors). All bars represent mean ± SD. Student's *t*‐test was used to compare the two experimental groups.

The microfluidic intracellular delivery platform effectively transfected NK cells with mRNA while minimizing adverse effects. Based on this success, we investigated the feasibility of using the Y‐hydroporator for CAR‐NK cell production. NK cells were hydroporated with mRNA encoding an anti‐CD19 CAR, which included a 4‐1BB co‐stimulatory endodomain, at a concentration of 50 µg mL⁻¹ (**Figure** **5**A(1)). To facilitate downstream in vitro cancer cell cytotoxicity assays, the cell density input for the microfluidic device was adjusted to 2 × 10⁷ cells mL⁻¹. Following hydroporation, the CAR mRNA‐transfected NK cells were incubated for 18 h at 37 °C. Anti‐CD19 CAR expression was evaluated by staining both negative control and hydroporated NK cells with PE‐conjugated CD19 antigen (Figure 5B). Approximately 74% of hydroporated NK cells expressed anti‐CD19 CAR, compared with the negative control group. As opposed to electroporation, the Y‐hydroporator demonstrated superior and more consistent CAR mRNA transfection across donors. Specifically, the Y‐hydroporator achieved ≈2.5‐fold higher transfection efficiency than electroporation, as shown in Figure 5C.

To confirm the cytotoxic capability of the CAR‐NK cells produced by the microfluidic device, we conducted an in vitro cytotoxicity assay (Figure 5A (3)).^[^ ^29^ ^]^ Anti‐CD19 CAR‐NK cells and control NK cells were co‐incubated with CD19‐positive Raji cells, a Burkitt's lymphoma cell line, at effector‐to‐target ratios of 2:1, 1:1, and 0.5:1 for 4 h. After co‐incubation, 7‐AAD staining was performed to detect the specific lysis of PE cell tracer‐stained Raji cells. Fluorescence microscopy (Figure 5D) revealed Raji cell apoptosis at a 2:1 effector‐to‐target ratio when co‐incubated with CAR‐NK cells. Quantitative flow cytometry analysis showed that CAR‐NK cells exhibited up to a 20% improvement in cytotoxicity against Raji cells compared with control NK cells, even at lower effector‐to‐target ratios (Figure 5E). In contrast, control NK cells displayed baseline cytotoxicity against Raji cells due to innate immune responses, underscoring the significance of the observed enhancement in cytotoxicity.^[^ ^28^ ^]^ These findings demonstrate that the Y‐hydroporator efficiently produces CAR‐NK cells with considerable potential for NK cell‐based CAR therapies.

### 2.6 Next‐Generation NK Cell‐Based Immunotherapy

Beyond hematological malignancies, the next frontier for CAR immunotherapy is the treatment of solid tumors.^[^ ^30^ ^]^ Successful remission of solid tumors depends on the ability of NK cells to infiltrate the tumor microenvironment (TME) effectively. To address this, we sought to validate the ability of our microfluidic device to produce next‐generation NK cell‐based immunotherapies for solid tumor applications. Unlike T cells, NK cells lack antigen specificity^[^ ^13c^ ^]^ and instead rely on signals from activating and inhibitory receptors to mount immune responses against tumors or infected cells. For example, MHC class I molecules on normal cells engage inhibitory receptors on NK cells, enabling selective targeting of MHC class I‐negative tumor cells. However, tumor cells in the TME often evade immune surveillance by upregulating human leukocyte antigen (HLA)‐E, a subtype of MHC class I, in response to interferon‐gamma secreted by NK cells. Strategies to enhance NK cell cytotoxicity by blocking the NKG2A inhibitory receptor have been explored.^[^ ^31^ ^]^ To this end, we aimed to produce NKG2A‐knockout NK cells using CRISPR/Cas9 ribonucleoproteins (RNPs) delivered via the Y‐hydroporator. This process targeted the NKG2A‐encoding killer cell lectin‐like receptor C1 (*KLRC1*) and evaluated the resulting cytotoxicity.

**Figure 6:**
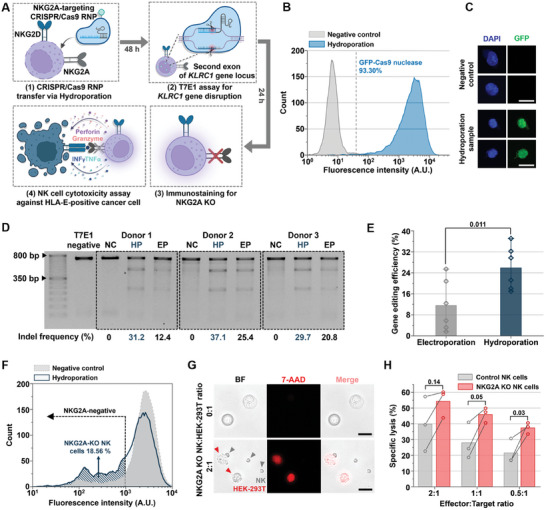
Next‐generation NK cell‐based immunotherapy via CRISPR/Cas9 RNP delivery. A) Schematic of inhibitory receptor knockout (KO) using CRISPR/Cas9 delivery. B) Fluorescence intensity histograms of GFP‐conjugated Cas9 protein delivery for the negative control and hydroporated NK cells after 24 h (*N*_cell_ = 5000 per sample). C) Confocal microscopy images of NK cells stained with DAPI and GFP following GFP‐conjugated Cas9 protein delivery (scale bar: 10 µm). D) Gel image displaying T7E1 assay results 48 h post‐delivery of CRISPR/Cas9 RNP with sgRNA #1 (NC: negative control, HP: hydroporation, EP: electroporation). E) Editing efficiency of the *KLRC1* gene using CRISPR/Cas9 RNP delivered via electroporation and hydroporation (*N* = three independent donors). F) Fluorescence intensity histograms showing PE‐conjugated NKG2A antibody immunostaining for the negative control and hydroporated NK cells after 96 h (*N*_cell_ = 5000 per sample). G) Microscopy images of bright‐field and fluorescence showing 7‐AAD staining for cell death assessments (scale bars: 25 µm). H) Specific lysis of HEK‐293T cells co‐incubated with control or NKG2A‐knockout NK cells (*N* = three independent donors). All bars represent mean ± SD. Student's *t*‐test was used to compare the two experimental groups.

To knockout *KLRC1*, we delivered CRISPR/Cas9 RNPs and performed cell cytotoxicity assays, as illustrated in **Figure** **6**A. Prior to introducing the RNPs into NK cells, we assessed the platform's ability to deliver large (≈160 kDa) Cas9 proteins. Following GFP‐conjugated Cas9 protein delivery into NK cells, flow cytometry analysis after 24 h revealed a high delivery efficiency of ≈93% (Figure 6B). Confocal microscopy confirmed nuclear localization of the Cas9 protein (Figure 6C), indicating the feasibility of genome editing using this method.

Subsequently, CRISPR/Cas9 RNPs, consisting of Cas9 protein (50 µg per 1 × 10⁷ cells) and single guide RNA (sgRNA) targeting *KLRC1* in a 2.5 molar excess, were delivered to the NK cells (Figure 6A(1)). Two sgRNAs with different sequences^[^ ^31b^ ^]^ were designed and delivered (Figure S3, Supporting Information). A T7 endonuclease 1 (T7E1) assay conducted 48 h post‐delivery quantified gene‐editing efficiency and evaluated non‐homologous end joining (NHEJ) resulting from target gene cleavage (Figures 6D and S3B (Supporting Information) for sgRNA #1 and #2 T7E1 results, respectively). Hydroporated NK cells achieved up to 37% gene‐editing efficiency for sgRNA #1. Compared to electroporation, a standard CRISPR‐mediated genome engineering method, hydroporated NK cells from three independent donors demonstrated superior editing efficiency (Figure 6E), suggesting that our platform is an effective alternative. Immunostaining performed 96 h after RNP delivery verified the knockout of the NKG2A receptor. Approximately 18.56% of hydroporated NK cells lacked NKG2A expression (Figure 6F). Considering the gene editing efficiency indicated by the T7E1 assay, the observed NKG2A‐negative population was lower than expected, likely due to the long NKG2A half‐life.^[^ ^32^ ^]^

After confirming successful NKG2A knockout, we conducted an in vitro cytotoxicity assay^[^ ^29^ ^]^ against HLA‐E‐positive cells. HEK‐293T cells, which express HLA‐E on the cell membrane,^[^ ^33^ ^]^ were used as a solid tumor model to evaluate the cytotoxicity of NKG2A‐knockout NK cells.^[^ ^34^ ^]^ Control and NKG2A‐knockout NK cells were co‐incubated with HEK‐293T cells at effector‐to‐target ratios of 2:1, 1:1, and 0.5:1 for 4 h. Specific lysis of carboxyfluorescein succinimidyl ester (CFSE)‐labeled HEK‐293T cells was assessed using 7‐AAD staining. Fluorescence microscopy revealed significant cell death in HEK‐293T cells co‐incubated with NKG2A knockout NK cells (Figure 6G). Flow cytometry analysis showed that NKG2A‐knockout NK cells exhibited a 21% increase in cytotoxicity compared to control NK cells, even at lower effector‐to‐target ratios (Figure 6H). These findings highlight the potential of our microfluidic platform to transform NK cell‐based immunotherapy through CRISPR‐mediated genome editing.

## 3 Conclusion

This study presents a microfluidic intracellular delivery device designed to overcome the limitations of conventional methods for producing NK cell‐based immunotherapies, such as high costs, mutagenic risks, impaired cell functionality, and low transfection efficiency. The Y‐junction hydroporator achieves a high processing rate of 1–2 × 10^6^ cells min^−1^ and approximately 90% transfection efficiency for EGFP mRNA, even at low concentrations. NK cells processed with this device maintained viability for up to six days and preserved their biomarker phenotypes, including CD56 and CD94. Furthermore, the device successfully delivered CAR‐encoding mRNA and CRISPR/Cas9 RNPs to NK cells, resulting in enhanced cytotoxicity. These findings highlight the potential of this microfluidic platform to generate NK cell‐based immunotherapies with improved therapeutic efficacy compared to conventional electroporation methods.

While this study employed a single‐channel design, future developments will focus on channel parallelization and automation to increase cell‐processing rates and enable the mass production of off‐the‐shelf allogeneic cell‐based immunotherapies. For instance, an 8‐channel parallelization system could prepare ≈1 billion NK cells in just one hour. Additionally, this implementation, along with reduced cargo consumption, would expand the device's applications to include in vivo immunotherapy and *ex vivo* gene therapies, such as those targeting sickle cell disease.

It is important to note that the efficiency levels reported here may appear lower than those in other studies due to the significant influence of various factors, including the cell source (peripheral blood *vs*. cord blood NK cells), cell state (naïve *vs*. activated), timing of tests, preparation protocols (e.g., feeder cell types, activation, and expansion methods), characterization methods, test solutions (media *vs*. specialized buffers), and user variability. These variables are especially pronounced in NK cell studies, which makes the process more sensitive and complex.^[^ ^35^ ^]^

Given its versatility in delivering diverse cargo types, such as mRNA and CRISPR systems, this platform has the potential to enable the delivery of genes encoding solid tumor‐specific CARs^[^ ^30a^ ^]^ or other receptors such as CD16 to enhance antibody‐mediated cytotoxicity by NK cells.^[^ ^28^ ^]^ Moreover, this platform could be adapted for use with other immune cell types, such as macrophages, thereby significantly broadening the scope of cell‐based immunotherapies. In conclusion, the microfluidic Y‐hydroporator demonstrates substantial potential as an advanced platform for next‐generation cell‐based therapies.

## 4 Experimental Section

### Preparation of Primary Immune Cells and Cancer Cell Lines

PBMCs were obtained with Institutional Review Board (IRB) approval (NK and γδ T cells: E2306/001‐003; αβ T cells: SMC 2021‐01‐091). To activate and expand primary NK cells in vitro, PBMCs were co‐cultured with 100 Gy gamma‐irradiated K562 cells (Gammacell 3000 Elan, Best Theratronics, Canada) in a 24‐well plate (SPL Life Sciences, Republic of Korea). The cells were resuspended in RPMI‐1640 medium (Corning, USA) supplemented with 10% fetal bovine serum (FBS, Gibco, Thermo Fisher Scientific, USA), 1% penicillin‐streptomycin (Gibco, Thermo Fisher Scientific, USA), 5 mM L‐glutamine (Gibco, Thermo Fisher Scientific), 10 IU mL^−1^ animal‐free recombinant human IL‐2 (PeproTech, USA), 100 µg mL^−1^ animal‐free recombinant human IL‐18 (MBL International, USA), and 10 µg mL^−1^ animal‐free recombinant human IL‐21 (PeproTech).^[^ ^36^ ^]^ After one week, irradiated K562 cells were added to reactivate the NK cells. NK cells were cultured at a density of 2 × 10^6^ cells mL^−1^ for up to 20 days in the presence of 200 IU mL^−1^ animal‐free recombinant human IL‐2 (PeproTech) and 0.5 µL mL^−1^ animal‐free recombinant human IL‐15 (PeproTech).

CD3‐positive cells were isolated from PBMCs using a MACS separator (Miltenyi Biotec, Germany) and cultured to activate and expand primary αβ T cells in vitro. The isolated T cells were resuspended in X‐VIVO 10 serum‐free hematopoietic cell medium (Lonza, Switzerland) supplemented with 25 µL mL^−1^ ImmunoCult™ Human CD3/CD28 T Cell Activator (STEMCELL Technologies, Canada) and 100 IU mL^−1^ animal‐free recombinant human IL‐2 at a starting density of 1 × 10⁶ cells mL^−1^ in a 24‐well plate. The αβ T cells were cultured for up to 13 days.

For γδ T cell preparation, Vδ2 T cells were activated and expanded from PBMCs. Vδ2 T cells were cultured at a density of 1.5 × 10⁶ cells mL^−1^ in RPMI‐1640 medium supplemented with 200 IU mL^−1^ animal‐free recombinant human IL‐2 and 10 µM zoledronate (Sigma Aldrich, Merck, Germany). The cells were cultured for up to 10 days.

Raji and HEK‐293T cells were obtained from the American Type Culture Collection (ATCC, USA). These cells were cultured in RPMI‐1640 medium supplemented with 10% FBS and 1% penicillin‐streptomycin in T75 cell culture flasks (SPL Life Sciences).

### Microfluidic Device Fabrication and Operation

The microfluidic channels were designed using AutoCAD (Autodesk, USA) and fabricated through deep reactive‐ion etching (EPG, Republic of Korea) on a 4‐inch wafer. PDMS channels were then produced using soft lithography with the Sylgard 184 elastomer kit (Dow, USA). The PDMS channels were treated with an O_2_ plasma system (CUTE, Femto Science Inc., Republic of Korea) to form a PDMS‐glass microfluidic chip.

For hydroporation, 1 and 3–5 mL Luer‐Lok syringes (BD, USA) for core and sheath flows, respectively, were connected to the microfluidic chip via PEEK tubing. Core and sheath flows were injected using two syringe pumps (PHD 2000, Harvard Apparatus, USA).

Cell processing was visualized using a Zeiss Axio Observer A1 inverted microscope (Carl Zeiss, Germany) and captured with a Phantom VEO710L high‐speed camera (Vision Research, USA). High‐speed imaging at 580 000 fps was used to observe the hydrodynamic elongation of NK cells. Clogging was quantified using high‐speed microscopic images, and clogging‐free scores were calculated. The clogging‐free score ranged from 100 to 0, with 25 points deducted for each 15‐second clogging interval. A score of 100 indicated uninterrupted flow throughout the processing period, while experiments halted by clogging were assigned a score of zero.

### Primary Immune Cell Engineering

For FITC‐dextran (Sigma‐Aldrich) delivery, the core flow consisted of cells suspended in growth media at a density of 1 × 10⁷ cells mL^−1^ along with cargos. The sheath flow was composed of Dulbecco's phosphate‐buffered saline (DPBS; Cytiva, USA). Following hydroporation, cells were washed with DPBS and resuspended in growth media for incubation at 37 °C.

For mRNA or CRISPR/Cas9 RNP transfection into NK cells, the core flow contained cells and cargos suspended in serum‐reduced Opti‐MEM (Gibco, Thermo Fisher Scientific) at densities ranging from 1 to 2 × 10⁷ cells mL^−1^. The sheath flow comprised X‐VIVO 10 serum‐free hematopoietic cell medium. Hydroporated cells were immediately transferred to prewarmed growth media and incubated at 37 °C. A Neon transfection system (Invitrogen, Thermo Fisher Scientific) was used to electroporate NK cells at 1800 V for 20 ms with a single pulse, following a customized device operation recipe from STEMCELL Technologies (Canada).

CleanCap EGFP mRNA was procured from Trilnk Biotechnologies (USA). Anti‐CD19 CAR mRNA was synthesized using a standard in vitro transcription method from a linearized vector template containing a second‐generation CAR construct (CD3ζ and 4‐1BB). CAR mRNA synthesis utilized the mMESSAGE mMACHINE™ T7 transcription kit (Invitrogen), replacing N1‐methylpseudouridine (TriLink Biotechnologies) to minimize immunogenicity. The synthesized mRNA was purified using the MEGAclear™ Transcription Clean‐Up Kit (Invitrogen).

For *KLRC1* or *KLRC1*‐targeting CRISPR/Cas9 RNP delivery, 23 guide RNA (gRNA) candidates targeting the second exon were evaluated for on‐ and off‐target scores using the CRISPR‐Cas9 gRNA checker (IDT Technologies, USA). The highest‐scoring gRNAs—sgRNA #1 (5’‐TGAACAGGAAATAACCTATG‐3’) from the literature^[^ ^31^, ^37^ ^]^ and sgRNA #2 (5’‐TTGAAGGTTTAATTCCGCAT‐3’)—were selected for NKG2A knockout. The Cas9 protein (Alt‐R™ S.p. Cas9 V3 and Alt‐R™ S.p. Cas9‐GFP V3, IDT Technologies) was initially suspended in a glycerol solution and underwent media exchange with Opti‐MEM using Pierce™ protein concentrators PES (Thermo Fisher Scientific) to optimize *Re* without affecting the viscosity of the cell suspension media. The Cas9 protein solution and sgRNA (Alt‐R CRISPR‐Cas9 sgRNA, IDT Technologies) were mixed and incubated at room temperature for 10 min to form RNP.

### Flow Cytometry Assay

Flow cytometry (Guava easyCyte, Luminex, USA) was performed to evaluate delivery and transfection efficiency, cell viability, and biomarker phenotype. Cells were stained with 7‐AAD (Invitrogen), diluted 1:50 in flow cytometry staining buffer (Invitrogen), to assess viability. After a 3‐minute incubation at room temperature, the cells were directly analyzed via flow cytometry. Delivery efficiency was quantified using the top 5% of fluorescence signals from the positive control group, while transfection efficiency was determined from the top 1% of the negative control group. Relative viability was calculated by dividing the viability of engineered NK cells by that of control NK cells.

For the cytotoxicity assay, target cells were stained with 1 µM reagent from the CellTrace™ Yellow Cell Proliferation Kit (Invitrogen) or eBioscience™ CFSE (Invitrogen). At a density of 5 × 10⁶ cells mL^−1^, cells were incubated in the dark for 10 min at room temperature. The cells were then washed with cold complete medium and resuspended for co‐incubation with effector cells. FITC, GFP, and CSFE detection were performed using a 488 nm laser and a 525/30 fluorescence filter, while 7‐AAD detection used a 695/50 fluorescence filter.

To evaluate biomarker phenotype, PE‐conjugated monoclonal antibodies against CD56 and CD94 (Invitrogen), and NKG2A (Miltenyi Biotec) were used for immunofluorescence staining of NK cells. First, 5 µL of antibodies were mixed with cells suspended in 100 µL of flow cytometry staining buffer and incubated for 20 min at 4 °C. Cells were subsequently washed with DPBS before flow cytometric analysis. NKG2A staining was performed after fixation and permeabilization using the eBioscience™ Intracellular Fixation & Permeabilization Buffer Set (Thermo Fisher Scientific).

Extracellular expression of anti‐CD19 CAR was detected via immunofluorescence staining using PE‐labeled CD19 protein (AcroBiosystems, USA). NK cells (2 × 10⁴) were stained with CD19 protein at 4 °C for 10 min, followed by two washes with DPBS before flow cytometry analysis. A 488 nm laser and 575/27 filter were used for PE fluorescence detection. All data were acquired using Guava InCyte 3.3 software.

### Confocal Microscopy

DAPI‐stained cells were observed using a Nikon C1 Plus laser‐scanning confocal microscope (Nikon, Japan). Delivered or transfected cells were fixed with 4% formaldehyde and stained with DAPI (Invitrogen) according to the manufacturer's protocol. Processed cells were adhered to poly‐L‐lysine‐coated glass slides and covered with mounting solution using a Cytospin–TXT3 system (Nasco Medilab, Republic of Korea).

### T7E1 Assay

Genomic DNA was extracted using QuickExtract™ DNA Extraction Solution (LGC Biosearch Technologies, United Kingdom) and a T100 Thermal Cycler (Bio‐Rad, USA). DNA was quantified using a NanoDrop system (Thermo Fisher Scientific). A polymerase chain reaction (PCR) mixture was prepared, including Quick Taq™ HS DyeMix (Toyobo, Japan) and primers for approximately 800 bp amplification (sgRNA #1: 5’‐CTTCTCTGGAGCTGATGGTAAAT‐3’, 5’‐CAATACTCGTTCTCCACCTCAC‐3’; sgRNA #2: 5’‐GTTACCACAGAGGCCATTAAGA‐3’, 5’‐CAATACTCGTTCTCCACCTCAC‐3’). PCR products were purified using the Monarch PCR & DNA Cleanup Kit (New England Biolabs, USA). After annealing, T7 Endonuclease I (New England Biolabs) was used to digest heteroduplexes formed by NHEJ. Digestion products were visualized using an iBright™ CL750 Imaging System (Invitrogen) following DNA electrophoresis (Wide Mini‐Sub Cell GT; PowerPac™, Bio‐Rad).

### Data Processing and Analysis

Images from high‐speed microscopy, agarose gel electrophoresis, and fluorescence analyses were processed using ImageJ (https://imagej.net/ij/) or MATLAB (MathWorks, USA). Data were replotted with Origin Pro (OriginLab, USA). Figures 1, 5, and 6A were created using BioRender.com.

## Conflict of Interest

The authors declare the following competing financial interests: A.J.C. has a financial interest in MxT Biotech, which is commercializing the platform presented in this work.

## Supporting information



Supporting Information

Supplemental Movie 1

Supplemental Movie 2

## Data Availability

The data that support the findings of this study are available from the corresponding author upon reasonable request.
