# Supplementary material for: Advancing Allogeneic NK Cell Immunotherapy through Microfluidic Gene Delivery
Source: Adv Sci (Weinh). 2025 Mar 7;12(17):2412544. doi: 10.1002/advs.202412544 (PMC12061328; doi:10.1002/advs.202412544)
Supplement: Supplementary file 1 — Supporting Information [file ADVS-12-2412544-s001.docx]

**Supporting Information**

**Advancing Allogeneic NK Cell Immunotherapy through**

**Microfluidic Gene Delivery**

Hyelee Kim^1,2^, Mujin Lee^3^, Bohwa Han^3^, Jinho Kim^4^, Duck Cho^4,5^, Junsang Doh^3^, and
Aram J. Chung^1,2,6,7*^

^1^Department of Bioengineering, Korea University, Seoul, 02841, Republic of Korea

^2^Interdisciplinary Program in Precision Public Health (PPH), Korea University, Seoul, 02841, Republic of Korea
^3^Department of Materials Science and Engineering, Seoul National University, Seoul, 08826, Republic of Korea

^4^Department of Health Sciences and Technology, SAIHST, Sungkyunkwan University, Seoul, 06355, Republic of Korea

^5^Department of Laboratory Medicine and Genetics, Samsung Medical Center, Sungkyunkwan University School of Medicine, Seoul, 03063, Republic of Korea
^6^School of Biomedical Engineering, Korea University, Seoul, 02841, Republic of Korea

^7^MxT Biotech, Seoul, 04785, Republic of Korea

^*^Corresponding authors [ac467@korea.ac.kr](mailto:ac467@korea.ac.kr)

**
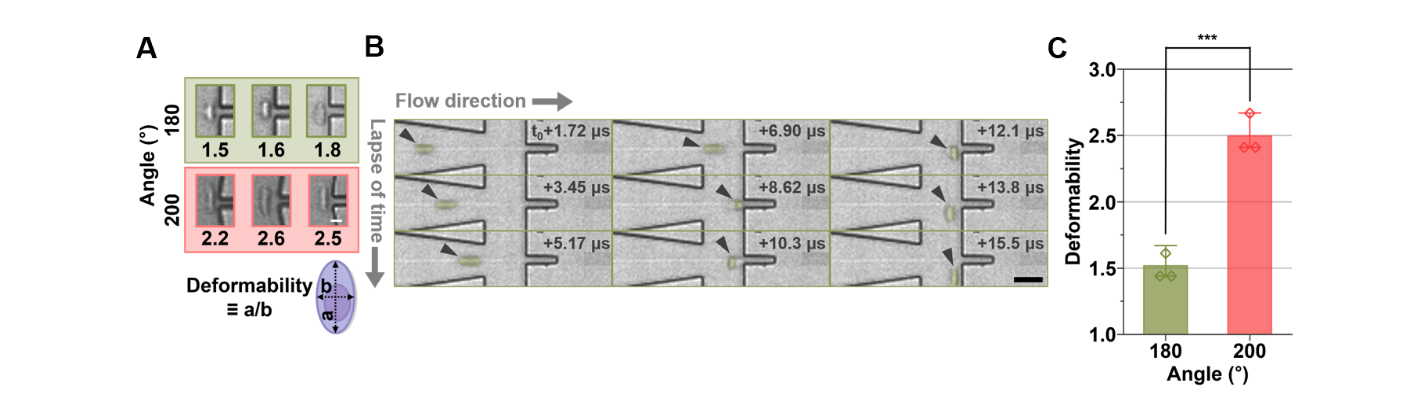
**

**Figure S1. NK cell deformability and elongation time. (A)** Microscopy images showing NK cell deformability at the stagnation point, depending on the junction angle. (**B)** High-speed microscopy images illustrating the hydrodynamic elongation of NK cells at a junction angle of 180°. **(C)** Deformability of NK cells at stagnation point. All bars represent the mean ± standard deviation (SD). *** indicates a *P*-value below 0.001. The Student’s *t*-test was used to compare two experimental groups.


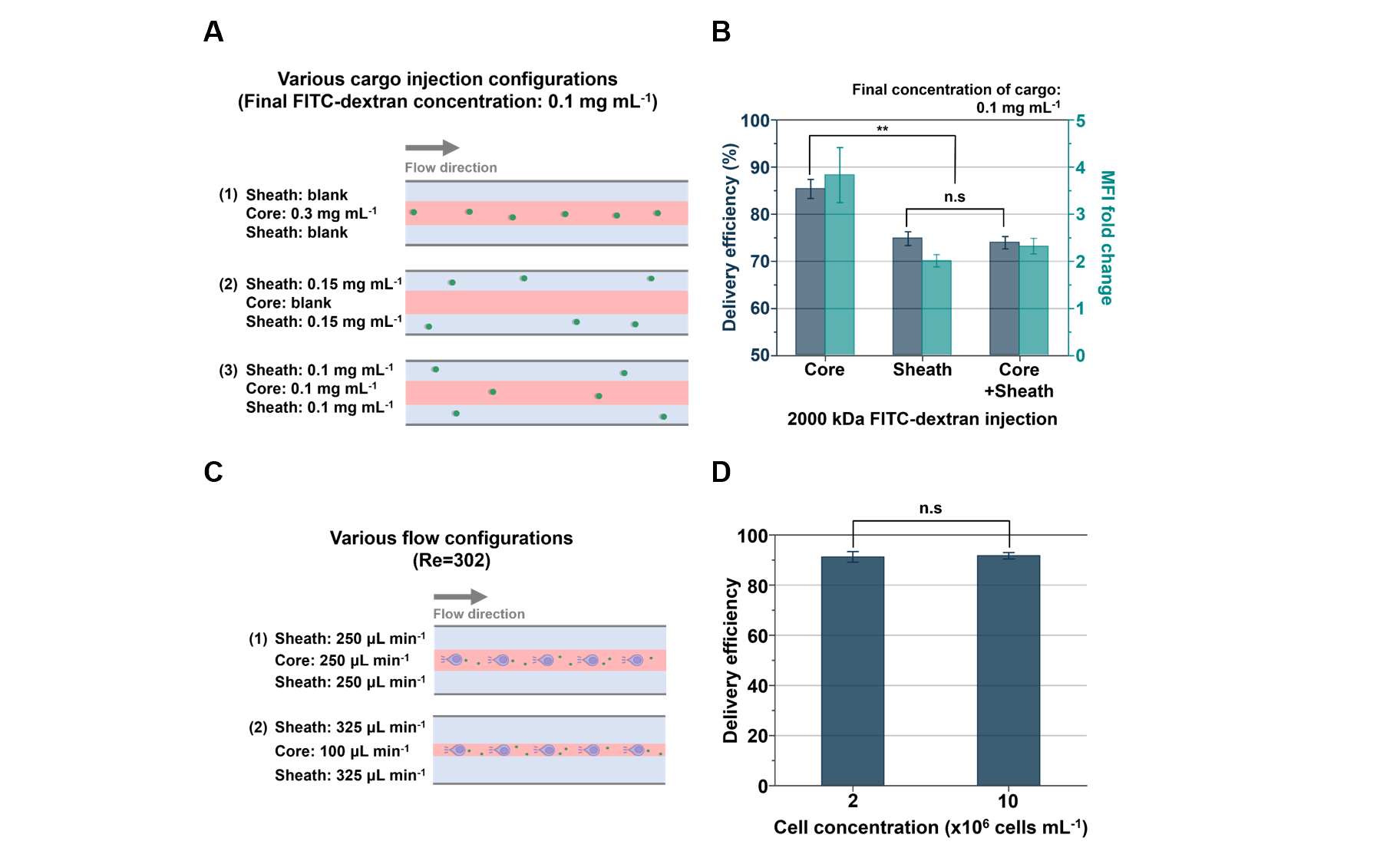


**Figure S2. Characterization of the workflow.** **(A)** Schematic representation of various cargo injection configurations. **(B)** Delivery efficiency and fold change in mean fluorescence intensity (MFI) for 2,000 kDa FITC-dextran delivery into NK cells using different cargo injection configurations (*N* = primary NK cells from three independent human donors). **(C)** Schematic depiction of two core and sheath flow rate distributions at the same Reynolds number (*Re*). **(D)** Delivery efficiency of 2,000 kDa FITC-dextran into NK cells with different flow rate configurations and cell concentrations (*N* = three independent donors). All bars represent the mean ± SD. n.s indicates no significant difference, and ** represents a *P*-value below 0.01. Multiple comparisons were performed using one-way analysis of variance (ANOVA), and the Student’s *t*-test was used for comparisons between two experimental groups.


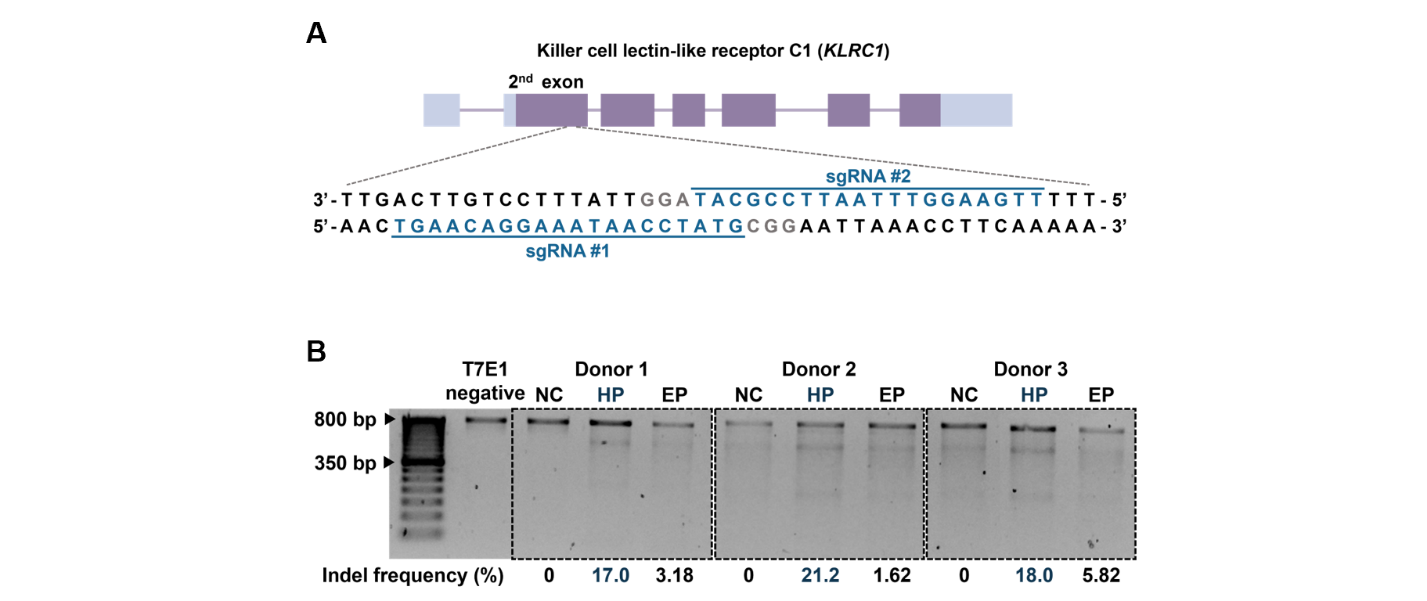


**Figure S3. sgRNA targeting *KLRC1*. (A)** Schematic of two sgRNAs targeting the second exon of the KLRC1 gene. **(B)** Gel image from the T7E1 assay performed 48 h after the CRISPR/Cas9 RNP delivery with sgRNA #2 (NC: negative control, HP: hydroporation, EP: electroporation).
